# Naturally occurring Ehrlichia chaffeensis infection in coyotes from Oklahoma.

**DOI:** 10.3201/eid0605.000505

**Published:** 2000

**Authors:** A A Kocan, G C Levesque, L C Whitworth, G L Murphy, S A Ewing, R W Barker

**Affiliations:** Oklahoma State University, Stillwater, Oklahoma 74078, USA.

## Abstract

A nested polymerase chain reaction assay was used to determine the presence of Ehrlichia chaffeensis, E. canis, and E. ewingii DNA in blood samples of free-ranging coyotes from central and northcentral Oklahoma. Of the 21 coyotes examined, 15 (71%) were positive for E. chaffeensis DNA; none was positive for E. canis or E. ewingii. Results suggest that E. chaffeensis infections are common in free-ranging coyotes in Oklahoma and that these wild canids could play a role in the epidemiology of human monocytotropic ehrlichiosis.


### Naturally Occurring *Ehrlichia chaffeensis* Infection in Coyotes from Oklahoma

**Alan Kocan, Gena Crowder Levesque, Lisa C. Whitworth, George L. Murphy, Sidney A. Ewing, and Robert W. Barker**
Oklahoma State University, Stillwater, Oklahoma, USA

A nested polymerase chain reaction assay was used to determine the presence of *Ehrlichia chaffeensis, E. canis,* and *E. ewingii* DNA in blood samples of free-ranging coyotes from central and northcentral Oklahoma. Of the 21 coyotes examined, 15 (71%) were positive for *E. chaffeensis* DNA; none was positive for *E. canis* or *E. ewingii*. Results suggest that *E. chaffeensis* infections are common in free-ranging coyotes in Oklahoma and that these wild canids could play a role in the epidemiology of human monocytotropic ehrlichiosis.

Human monocytotropic ehrlichiosis, a tick-borne zoonosis caused by the rickettsial pathogen *Ehrlichia chaffeensis* (Rickettsiales: Ehrlichieae), occurs primarily in the southern, southcentral, and mid-Atlantic regions of the United States (1,2). The principal vector is the lone star tick, *Amblyomma americanum* (L.), and associations between the presence of the tick and the occurrence of human ehrlichiosis have been documented (1). The principal wildlife reservoir is the white-tailed deer *(Odocoileus virginianus)* (3,4). Indeed, site-specific geographic and temporal associations have been made between the presence of *A. americanum* and *E. chaffeensis* antibodies in deer (5,6). No other wildlife species has been incriminated in the epidemiology of this disease, although serologic reactivity was detected in free-ranging raccoons *(Procyon lotor)* and opossums (*Didelphis virginianus*) from Georgia (5) and white-footed mice (*Peromyscus leucopus*) from Connecticut (7). Additionally, red foxes (*Vulpes vulpes*) have been shown to be susceptible to infection under experimental conditions (8). Although some rodents have been experimentally infected with this pathogen (9), research findings about natural infections in wild rodent populations have been inconsistent (7,10). Domestic dogs are susceptible to both natural and experimental *E. chaffeensis* infections (11-13).

Address for correspondence: A. Alan Kocan, Department of Veterinary Pathobiology, College of Veterinary Medicine, Oklahoma State University, Stillwater, OK 74078; fax: 407-744-8263; e-mail: aak4453@okstate.edu.

### Methods and Study Design

To determine whether free-ranging coyotes (*Canis latrans*) serve as a reservoir host for *E. chaffeensis, E. canis*, or *E. ewingii*, we used a nested polymerase chain (PCR) assay to survey for the presence of DNA of these organisms in blood samples from 21 free-ranging coyotes from central and northcentral Oklahoma. Coyotes were obtained as part of animal damage control (U.S. Department of Agriculture) from an area in the established range of *A. americanum* (14,15), in which *E. chaffeensis* was endemic in deer and *E. chaffeensis, E. canis,* and *E. ewingii* had been found in dogs (13,16). Immediately after the coyotes were shot, EDTA-anticoagulated whole blood was collected for isolation of DNA for PCR assay. Blood samples were stored at 4°C until processing. DNA was isolated from whole blood (200 µl) with the QIAamp Blood Kit (Qiagen, Santa Clarita, CA), according to the manufacturer's instructions.

Purified DNA from each blood sample was tested in four PCR amplifications by using primers HE1, HE3, EE5, and ECAN5 (12,13,17); reaction conditions are described in Figure 1. For DNA sequencing, PCR reactions were performed, and products were separated by agarose gel electrophoresis. Bands were stabbed multiple times with sterile pipet tips, which were placed into PCR reaction mix as template (19). PCR reactions were pooled and purified by using Qiagen Qiaquick PCR purification kit, according to manufacturer's instructions. DNA was sequenced at the Oklahoma State University

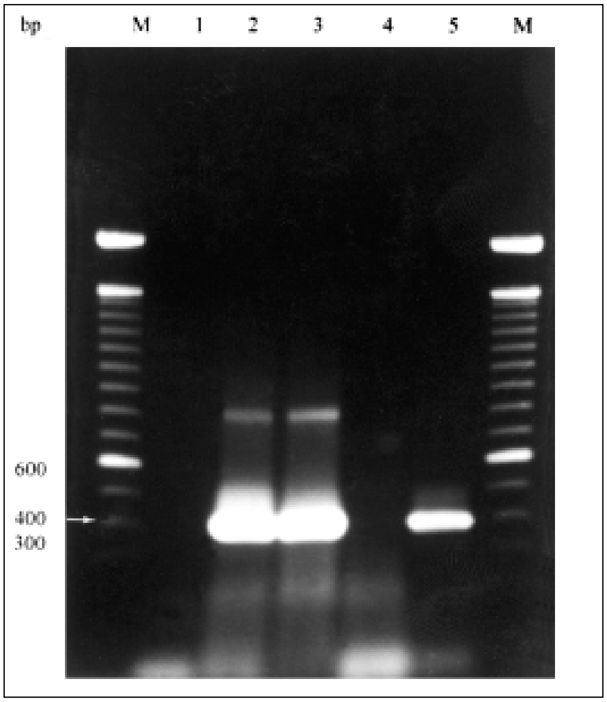

Figure 1. Agarose gel electrophoresis of results of PCR amplification of *Ehrlichia chaffeensis* nss rRNA gene from whole blood samples of coyotes numbers 9-11.* Lane 1= negative control (no DNA); Lane 2= coyote 9 (+); Lane 3= coyote 10 (+); Lane 4= coyote 11 (-); Lane 5 = positive control (*E. chaffeensis*-infected DH82 cells). M = 100-bp DNA ladder (Life Technologies, Rockville, MD).*

**Ehrlichia* forward primer ECC (5'-AGAACGAACGCTG-GCG GCAAGC-3') and *Ehrlichia* reverse primer ECB (5'-CGT ATTACCGCGGCTGCTGGCA-3') amplified all *Ehrlichia* spp (12,18). These reactions (50 µl) contained 10 µl of template DNA in 10 mM Tris-Cl (pH 8.3), 0.2 mM each deoxynucleoside triphosphate (dNTP), 2 mM MgCl2, 50 mM KCl, 0.5 µm each primer, and 1.25 U of Taq DNA polymerase (Promega Corporation, Madison, WI). A hot-start PCR was used in which each enzyme was added to reactions after an initial 3-min denaturation step at 94°C. Reactions consisted of 30 cycles of denaturation at 94°C for 1 min, annealing at 65°C for 2 min, and extension at 72°C for 2 min. Products of this reaction were used as template with species-specific primer sets for three nested reactions. Primers HE1 (5'-CA ATTGCTTATAACCTTTTGGTTATAAAT-3') and HE3 (5'-TA TAGGTACCGTCATTATCTTCCCTAT-3') (17) were used for *E. chaffeensis*-specific amplifications. Primers ECAN5 (5'-C AATTATTTATAGCCTCTGGCTATAGGA-3') (12,13) and HE3 were used for *E. canis*-specific amplifications, and primers EE5 (5'-(CAATTCCTAAATAGTCTCTGACTATT-TAG-3') (this study) and HE3 were used for *E. ewingii*-specific amplifications. Reactions (50 µl) contained 10 µl of the reaction product with ECC and ECB primers as template, and the remaining reaction components as above. A hot-start PCR was used in which the enzyme was added to reactions after an initial 3-min denaturation step at 94°C. Reactions with species-specific primers were in two stages. The first consisted of three cycles of denaturation at 94°C for 1 min, annealing at 55°C for 2 min, and extension at 72°C for 1.5 min. The second consisted of 37 cycles of denaturation at 92°C for 1 min, annealing at 55°C for 2 min, and extension at 72°C for 1.5 min. Distilled, deionized water served as a negative control. Positive control DNA samples were purified from *E. chaffeensis*-infected DH82 cells, blood from a dog experimentally infected with *E. canis*, and diluted general primer PCR reactions of synovial fluid from a dog experimentally infected with *E. ewingii.* To prevent contamination of samples, DNA purification, PCR master mix assembly, and amplifications were performed in separate rooms. Positive displacement pipetters and aerosol-free pipette tips were also used as further precautions.

Recombinant/DNA Protein Research Facility (Stillwater, OK) using an Applied Biosystems (Foster City, CA) 373A automated DNA sequencer. Sequences were analyzed with MacVector software (Oxford Molecular Group, Inc., Campbell, CA). A partial sequence (300 bp) from each end of the 390-bp amplified fragment was determined for both the *E. chaffeensis*-positive control and one positive coyote. The sequences obtained here were compared to those previously deposited in GenBank (13) to verify that *E. chaffeensis* DNA was amplified.

### Results and Discussion

Of the 21 coyotes tested, 15 were positive by PCR assay for *E. chaffeensis* (Figure 1); none was positive for *E. canis* or *E. ewingii.* To our knowledge, this is the first reported evidence of natural *E. chaffeensis* infection in a coyote and the first PCR-based evidence in a free-ranging mammal other than white-tailed deer. Although these findings do not question the importance of white-tailed deer in the endemic maintenance of *E. chaffeensis*, they do point to coyotes as potential reservoir hosts.

All stages of *A. americanum* feed readily on coyotes (14,20). Moreover, white-tailed deer and coyote populations overlap in much of the *E. chaffeensis–A. americanum* disease-endemic regions of the United States (1,21-24). Movement of these deer, as indicated by their home range (usually not exceeding 1.6 km [25]) is more restricted than that of coyotes (whose range may exceed 31 km [22]). These behavioral factors, and coyotes' apparent susceptibility to infection with *E. chaffeensis*, make them an ideal bridge species for the spread of this tickborne pathogen among wild species as well as a source of infection for ticks that may subsequently feed on other hosts, including humans and domestic animals.

The results of this study, although based on a limited number of free-ranging coyotes, suggest that in the geographic range of the study, coyotes likely play little or no role in the endemic maintenance or spread of other species of *Ehrlichia* that commonly parasitize domestic dogs or humans. Coyotes are susceptible to experimental infection with *E. canis* (26), and domestic dogs and ticks from Oklahoma have been shown to be naturally infected with both *E. canis* and *E. ewingii* as well as *E. chaffeensis* (13). In fact, *E. ewingii* DNA was recently identified from patients in Missouri, which expands the known host range of this organism, making it a newly emerging zoonosis of public health concern (27).

The occurrence of *E. ewingii* in domestic dogs and ticks in Oklahoma (13), the broad host range of *A. americanum* (its natural vector [14,23,28]), and the documented occurrence of *A. americanum* in both wild and domestic canids (13,20,24) suggest a potential for future cross-species transmission of this organism from domestic to wild and human hosts.

### Acknowledgments

We thank J. Lilley and D. Alley for their assistance in obtaining coyotes.

This research was supported in part by Animal Health Grant No. 1433 and project 3714 of the Agricultural Experiment Station and the College of Veterinary Medicine, Oklahoma State University.

Dr. Kocan is a professor in the Department of Veterinary Pathobiology at Oklahoma State University, College of Veterinary Medicine. His research interests include infectious and parasitic diseases of free-ranging wild animals and vector-borne diseases of wild, domestic, and human hosts.

### References

1. Eng TR, Harkess JR, Fishbein DB, Dawson JE, Greene CN, Rredus MA, et al. Epidemiologic, clinical and laboratory findings of human ehrlichiosis in the United States,1988. JAMA 1990;264:2251-8.
2. Dawson JE, Childs JE, Biggie KL, Moore C, Stallknecht DE, Shaddock J, et al. White-tailed deer as a potential reservoir for *Ehrlichia chaffeensis*, the etiologic agent of human ehrlichiosis. J Wildl Dis 1994;30:162-8.
3. Ewing SA, Dawson JE, Kocan AA, Barker RW, Warner CK, Panciera RJ, et al. Experimental transmission of *Ehrlichia chaffeensis* (Rickettsiales: Ehrlichieae) among white-tailed deer by *Amblyomma americanum* (Acari: Ixodidae). J Med Entomol 1995; 32:368-74.
4. Lockhart JM, Davidson WR, Dawson JE, Stallknecht DE, Howerth EW. Isolation of *Ehrlichia chaffeensis* from wild white-tailed deer (*Odocoileus virginianus*) confirms their role as natural reservoir hosts. J Clin Microbiol 1997;35:1681-6.
5. Lockhart JM, Davidson WR, Dawson JE, Stallknecht DE, Little SE. Natural history of *Ehrlichia chaffeensis* in the Piedmont physiographic province of Georgia. J Parasitol 1997;83:887-94.
6. Lockhart JM, Davidson WR, Dawson JE, Stallknecht DE. Temporal association of *Amblyomma americanum* with the presence of *Ehrlichia chaffeensis* reactive antibodies in white-tailed deer. J Wildl Dis 1995;31:119-24.
7. Magnarelli LA, Anderson AF, Stafford KC, Dumler JS. Antibodies to multiple tick-borne pathogens of babesiosis, ehrlichiosis, and Lyme borreliosis in white-footed mice. J Wildl Dis 1997;33:466-73.
8. Davidson WR, Lockhart JM, Stallknecht DE, Howerth EW. Susceptibility of red and gray foxes to infection by *Ehrlichia chaffeensis*. J Wildl Dis 1999;35:696-702.
9. Telford SR, Dawson JE. Persistent infection of C3H/HeJ mice by *Ehrlichia chaffeensis*. Vet Microbiol 1996;52:103-12.
10. Lockhart JM, Davidson WR, Stallknecht DR, Dawson JE. Lack of seroreactivity to *Ehrlichia chaffeensis* among rodent populations. J Wildl Dis 1998;34:392-6.
11. Dawson JE, Ewing SA. Susceptibility of dogs to infection with *Ehrlichia chaffeensis*, causative agent of human ehrlichiosis. Am J Vet Res 1992;53:1322-7.
12. Dawson JE, Biggie KL, Warner CK, Cookson K, Jenkens S, Levine JF, et al. Polymerase chain reaction evidence of *Ehrlichia chaffeensis*, an etiologic agent of human ehrlichiosis, in dogs from southeast Virginia. Am J Vet Res 1996;57:1175-9.
13. Murphy GL, Ewing SA, Whitworth LC, Fox JC, Kocan AA. A molecular and serologic survey of *Ehrlichia canis, E. chaffeensis*, and *E. ewingii* in dogs and ticks from Oklahoma. Vet Parasitol 1998;79:325-39.
14. Hair JA, Bowman JL. Behavioral ecology of *Amblyomma americanum* (L.). In: Sauer J, Hair JA, editors. Morphology, physiology, and behavioral biology of ticks. New York: Ellis Horwood Limited;1986. p. 406-27.
15. Cooley RA, Kohls GM. The genus *Amblyomma* (Ixodidae) in the United States. J Parasitol 1944;30:77-111.
16. Dawson JE, Warner CK, Baker V, Ewing SA, Stallknecht DE, Davidson WR, et al. *Ehrlichia*-like 16S rDNA from wild white-tailed deer (*Odocoileus virginianus*). J Parasitol 1996;82:52-8.
17. Anderson BE, Sumner JW, Dawson JE, Tzianabos T, Green CR, Olson JG, et al. Detection of the etiologic agent of human ehrlichiosis by polymerase chain reaction. J Clin Microbiol 1992;30:775-80.
18. Dawson JE, Stallknecht DE, Howerth EW, Warner C Biggie K, Davidson WR, et al. Susceptibility of white-tailed deer (*Odocoileus virginianus*) to infection with *Ehrlichia chaffeensis*, the etiologic agent of human ehrlichiosis. J Clin Microbiol 1994;32:2725-8.
19. Wilton SD, Lim L, Dye D, Laing N. Bandstab: a PCR-based alternative to cloning PCR products. BioTechniques 1997;22:642-5.
20. Kocan AA, Breshears M, Cummings C, Panciera RJ, Ewing SA, Barker RW. Naturally occurring hepatozoonosis in coyotes from Oklahoma. J Wildl Dis 1999;35:86-9.
21. Baker RH. Origin, classification, and distribution. In: Halls L, editor. White-tailed deer. ecology and management. Harrisburg (PA): Stackpole Books;1984. p. 1-18.

22. Nowak RM. Walker's mammals of the world (5th ed). Baltimore: The John Hopkins University Press;1991. vol. 2. p. 1068-70.
23. Patrick CD, Hair JA. White-tailed deer utilization of three different habitats and its influence on lone star tick populations. J Parasitol 1978;64:1100-6.
24. Bloemer SR, Zimmerman RH. Ixodid ticks on the coyote and gray fox at Land-Between-the-Lakes, Kentucky-Tennessee, and implications for tick dispersal. J Med Entomol 1988;25:5-8.
25. Morchonton RL, Hirth DH. Behavior. In: Halls L, editor. White-tailed deer: ecology and management. Harrisburg (PA): Stackpole Books;1984. p. 129-68.
26. Ewing SA, Buckner RG, Stringer BG. The coyote, a potential host for *Babesia canis* and *Ehrlichia* sp. J Parasitol 1964;50:704.
27. Buller RS, Arens M, Hmiel SP, Paddock CD, Sumner JW, Rikihisa Y, et al. *Ehrlichia ewingii*, a newly recognized agent of human ehrlichiosis. N Engl J Med 1999;341:148-55.
28. Anziani OS, Ewing SA, Barker RW. Experimental transmission of a granulocytic form of the Tribe Ehrlichieae by *Dermacentor variabilis* and A*mblyomma americanum* to dogs. Am J Vet Res 1990;51:929-31.
